# The First Lines of the Theory and Practice of Surgery, Including the Principal Operations

**Published:** 1845-04

**Authors:** 


*The first lines of the Theory and Practice of Surgery, including the principal operations.* By SAMUEL COOPER. *With notes and additions*, by WILLARD PARKER, M.D., &c. 2 vols. 8vo. New York: Samuel S. & William Wood. (From the publishers.)

This is a work so well known to the profession, that it is only necessary to announce a new edition, to ensure it a favorable reception. The preceding editions, edited by Dr. Stevens of New York, and Dr. M'Clellan of Philadelphia, had been for some time exhausted when this was issued. The notes and additions by Dr. Parker are judicious, valuable, and in strict accordance with the original plan of the work, and render it a very perfect work on the principles of surgery, in the present perfected state of the science. The mechanical part is well executed.—D. B.

